# Unexpected Compensatory Increase in *Shank3* Transcripts in *Shank3* Knock-Out Mice Having Partial Deletions of Exons

**DOI:** 10.3389/fnmol.2019.00228

**Published:** 2019-09-19

**Authors:** Chunmei Jin, Hyae Rim Kang, Hyojin Kang, Yinhua Zhang, Yeunkum Lee, Yoonhee Kim, Kihoon Han

**Affiliations:** ^1^Department of Neuroscience, College of Medicine, Korea University, Seoul, South Korea; ^2^Department of Biomedical Sciences, College of Medicine, Korea University, Seoul, South Korea; ^3^Division of National Supercomputing, Korea Institute of Science & Technology Information (KISTI), Daejeon, South Korea

**Keywords:** *Shank3*, knock-out mice, transcripts, compensation, exon

## Abstract

Genetic variants of the SH3 and multiple ankyrin repeat domains 3 (*SHANK3*) gene, which encodes excitatory postsynaptic core scaffolds cause numerous brain disorders. Several lines of *Shank3* knock-out (KO) mice with deletions of different *Shank3* exons have previously been generated and characterized. The different *Shank3* KO mouse lines have both common and line-specific phenotypes. Shank3 isoform diversity is considered a mechanism underlying phenotypic heterogeneity, and compensatory changes through regulation of *Shank3* expression may contribute to this heterogeneity. However, whether such compensatory changes occur in *Shank3* KO mouse lines has not been investigated in detail. Using previously reported RNA-sequencing analyses, we identified an unexpected increase in *Shank3* transcripts in two different *Shank3* mutant mouse lines (*Shank3B* and *Shank3ΔC*) having partial deletions of *Shank3* exons. We validated an increase in *Shank3* transcripts in the hippocampus, cortex, and striatum, but not in the cerebellum, of *Shank3B* heterozygous (HET) and KO mice, using qRT-PCR analyses. In particular, expression of the N-terminal exons 1–12, but not the more C-terminal exons 19–22, was observed to increase in *Shank3B* mice with deletion of exons 13–16. This suggests a selective compensatory activation of upstream *Shank3* promoters. Furthermore, using domain-specific Shank3 antibodies, we confirmed that the increased *Shank3* transcripts in *Shank3B* KO mice produced a small Shank3 isoform that was not detected in wild-type mice. Taken together, our results illustrate another layer of complexity in the regulation of *Shank3* expression in the brain, which may also contribute to the phenotypic heterogeneity of different *Shank3* KO mouse lines.

## Introduction

**Figure 1:**
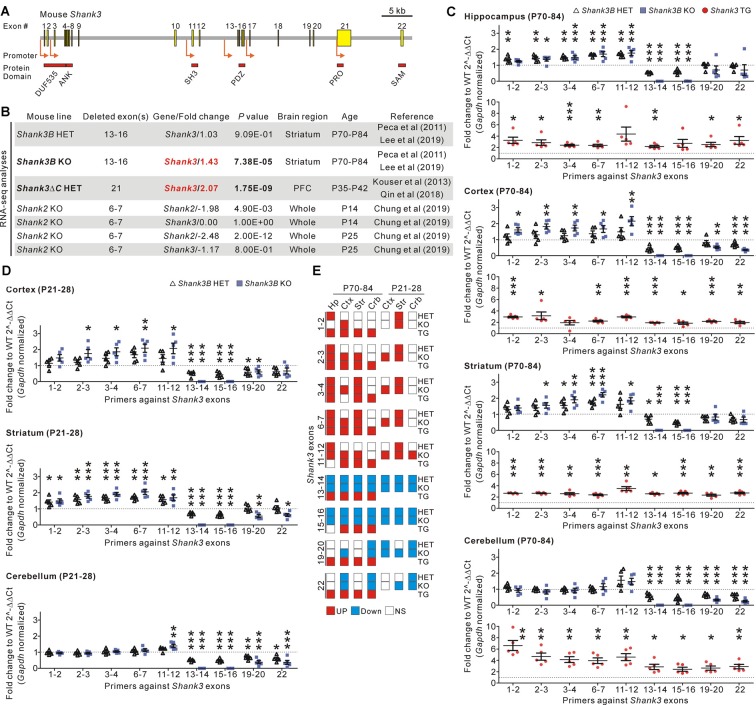
Identification and validation of increased *Shank3* transcript abundance in the brain regions of *Shank3* mutant mice with partial exon deletions. **(A)** Schematic diagram showing the structure of the mouse *Shank3* gene. The locations of the intragenic promoters and protein domains below their respective encoding exons are indicated. ANK, ankyrin repeat domain; DUF535, protein domain of unknown function 535; PDZ, postsynaptic density 95/discs large/zonula occludens 1 domain; PRO, proline-rich region; SAM; sterile alpha motif; SH3, SRC homology 3 domain. **(B)** Summary of changes in the* Shank3* and *Shank2* transcript levels obtained from the previously reported RNA-sequencing analyses of different *Shank3* and *Shank2* mutant mouse lines. P, postnatal day; PFC, prefrontal cortex. **(C)** qRT-PCR validation of *Shank3* transcript levels in the four brain regions of adult *Shank3B* heterozygous (HET) and knock-out (KO), and *Shank3* TG mice compared to their respective WT littermates (*n* = 5 animals per genotype). **(D)** qRT-PCR analysis of *Shank3* transcript levels in the cortex, striatum, and cerebellum of juvenile *Shank3B* HET and KO mice compared to the WT littermates (*n* = 5 animals per genotype). **(E)** Summary of the qRT-PCR analyses. Crb, cerebellum; Ctx, cortex; Hp, hippocampus; NS, not significant; Str, striatum. Data are presented as mean ± SEM. *P < 0.05; **P < 0.01; ***P < 0.001 [one-way analysis of variance (ANOVA) with Tukey’s pos*t*-test for WT, HET, and KO; unpaired two-tailed Student’s *t*-test for WT and TG].

Deletions, duplications, and various point mutations of the SH3 and multiple ankyrin repeat domains 3 (*SHANK3*) gene that encodes neuronal excitatory postsynaptic core scaffolds are causally associated with numerous brain disorders, including autism spectrum disorders, bipolar disorder, intellectual disability, and schizophrenia (Grabrucker et al., 2011; Monteiro and Feng, 2017). Previously, several *Shank3* mutant mouse models (i.e., knock-out (KO), knock-in, viral-mediated knock-down, and overexpression) mimicking conditions in patients, were generated and their neurobehavioral phenotypes were characterized in detail (Jiang and Ehlers, 2013; Monteiro and Feng, 2017). Specifically, more than ten different lines of *Shank3* KO mice having deletions of different* Shank3* exons were generated and mutant phenotypes both common and specific to certain lines were identified (Monteiro and Feng, 2017). The mouse *Shank3* gene has 22 exons and expresses several protein isoforms as a result of processing multiple intragenic promoters and alternative splicing (Wang et al., 2014; Figure 1A). Therefore, different subsets of Shank3 isoforms are disrupted in different *Shank3* KO mouse lines having partial deletions of exons, which can contribute to the phenotypic heterogeneity among the KO mouse lines.

Because of its crucial roles in synaptic development and function, *Shank3* gene expression, and Shank3 protein stability and interaction are tightly controlled by multiple mechanisms from the transcriptional to post-translational levels (Zhu et al., 2014; Choi et al., 2015; Kerrisk Campbell and Sheng, 2018; Wang et al., 2019). Therefore, in addition to isoform diversity, any compensatory changes in these *Shank3* regulatory mechanisms may also contribute to variable phenotypes in different *Shank3* KO mouse lines. However, whether such compensatory changes in regulation occur in any of the *Shank3* KO mouse lines has not yet been investigated in detail.

In this study, we identified and validated an unexpected increase in *Shank3* transcripts in the brain regions of *Shank3B* mice, in which exons 13–16 of the *Shank3* gene are targeted. This increase occurred in both heterozygous (HET) and KO mice. The increase was mainly observed from the N-terminal (1–12) *Shank3* exons in terms of the deleted exons in *Shank3B* mice, suggesting selective compensatory activation of upstream* Shank3* promoters. Furthermore, we confirmed that the upregulated *Shank3* transcripts produced a small Shank3 protein isoform in *Shank3B* KO brains. Our results reveal a novel compensatory change with respect to regulating *Shank3* expression in the brain, which may also contribute to the phenotypic heterogeneity of different *Shank3* KO mouse lines.

## Materials and Methods

### Mice

The enhanced green fluorescent protein (*EGFP*)-*Shank3* transgenic (TG), and *Shank3B* HET and KO mice used in this study have been described previously (Peca et al., 2011; Han et al., 2013b; Lee et al., 2017a; Lee B. et al., 2017). The mice were bred and maintained in a C57BL/6J (Japan SLC, Inc.) background according to the Korea University College of Medicine Research Requirements, and all the experimental procedures were approved by the Committee on Animal Research at the Korea University College of Medicine (KOREA-2016-0096). The mice were had access to water and food *ad libitum* and were housed at 4–6 mice per cage under a 12-h light-dark cycle at 18–25°C. For all experiments, only male mice were used, and WT control refers to the WT littermates of the TG or HET and KO mice.

### RNA Purification and qRT-PCR

Real-time quantitative reverse transcription PCR (qRT-PCR) was performed as described previously (Kim et al., 2016; Lee B. et al., 2017; Jin et al., 2018b). Briefly, total RNA was extracted from the brain regions of WT and *Shank3* TG as well as WT, *Shank3B* HET, and KO mice using an miRNeasy Mini Kit (Qiagen, #217004) according to the manufacturer’s instructions. 1.5 μg of total RNA was used for cDNA synthesis using an iScript™ cDNA Synthesis Kit (Bio-Rad, #170-8891). Target mRNAs were detected and quantified by a real-time PCR instrument (CFX96 Touch, Bio-Rad) using SYBR Green master mix (Bio-Rad, #170-8884AP). The results were analyzed using the comparative Ct method normalized against the housekeeping gene *Gapdh* (Han et al., 2013a). The primer sequences for real-time PCR are as follows:

Mouse *Shank3* (exons 1–2)

forward 5′ CGGACCTGCAACAAACGAAG 3′,
reverse 5′ TGTCCAGGTTAGGCGGGTAG 3′

Mouse *Shank3* (exons 2–3)

forward 5′ TCTGCGCCCTCAATCATAGC 3′,
reverse 5′ AGCTTTGCAAACTGCTTGTCA 3′

Mouse *Shank3* (exons 3–4)

forward 5′ GCGGAGAGTTTATGCCCAGA 3′,
reverse 5′ GGCCACCTTATCTGTGCTGT 3′

Mouse *Shank3* (exons 6–7)

forward 5′ TGGTTGGCAAGAGATCCAT 3′,
reverse 5′ TTGGCCCCATAGAACAAAAG 3′

Mouse *Shank3* (exons 11–12)

forward 5′ CAAGTTCATCGCTGTGAAGG 3′,
reverse 5′ TGTCGCATCTGCACTTCTTC 3′

Mouse *Shank3* (exons 13–14)

forward 5′ TCTTCCGCCACTACACTGTG 3′,
reverse 5′ AAAGCCAAACCCCTCATGGT 3′

Mouse *Shank3* (exons 15–16)

forward 5′ TTACACCCACACCTGCCTTC 3′,
reverse 5′ CACCATCCTCCTCGGGTTTC 3′

Mouse *Shank3* (exons 19–20)

forward 5′ ACATTGCAGATGCTGACTCG 3′,
reverse 5′ CAGATTTGGTCCGTGGAATC 3′

Mouse *Shank3* (exon 22)

forward 5′ AGTACCCCTTCGGGCTTCTA 3′,
reverse 5′ CAGACTCCAAACCCGATGTT 3′

Mouse* Gapdh*

forward 5′ GGCATTGCTCTCAATGACAA 3′,
reverse 5′ CCCTGTTGCTGTAGCCGTAT 3′

Specificity of each primer set was confirmed by examining the melting peaks of qRT-PCR reactions and the band size of PCR products from the reactions (Supplementary Figure S1).

### Western Blot Analysis

Whole lysate of the mouse brain tissue was prepared as previously described (Han et al., 2009, 2015; Zhang et al., 2018). Briefly, frozen mouse brain tissue was homogenized in RIPA buffer (50 mM Tris-HCl pH 8.0, 150 mM NaCl, 0.1% SDS, 1% Triton X-100, 0.5% sodium deoxycholate) with freshly added protease and phosphatase inhibitors (Sigma-Aldrich, #11836170001 and #4906837001, respectively). Protein concentration was measured using the Bradford Protein Assay (Bio-Rad, #500-0006). The lysate was heated in 1X NuPAGE LDS sample buffer (Thermo Fisher Scientific, #NP0007) containing 1X NuPAGE reducing agent (Thermo Fisher Scientific, #NP0004). From each sample, 20 μg of protein was loaded into 4%–15% Mini-PROTEAN TGX™ Precast Protein Gels (Bio-Rad, #4561084) for western blotting. The proteins were then transferred to a PVDF membrane (Millipore, #IPVH00010). The primary antibodies used for western blot analysis were Shank3 Ab#1 (aa 192–221) and Ab#2 (aa 529–558; kindly gifted by Prof. Eunjoon Kim, KAIST; Lee et al., 2015), Shank3 Ab#3 (aa 1431–1590, Santa Cruz, #sc-30193), and GAPDH (Cell Signaling, #2118S). Western blot images were acquired with the ChemiDoc Touch Imaging System (Bio-Rad) and quantified using ImageJ software.

## Results and Discussion

In recent RNA-sequencing analyses of the striatum of adult *Shank3B* HET and KO mice (Lee et al., 2019), we had unexpectedly observed significantly increased total *Shank3* transcripts in the KO striatum when compared to the WT striatum (Figure 1B). This raised our interest in the potential compensatory changes that occur in *Shank3* KO mouse lines. To understand whether this increase in *Shank3* transcripts was specific to the *Shank3B* KO line alone, we consulted another recently reported RNA-sequencing analysis (Qin et al., 2018) of the prefrontal cortex of *Shank3ΔC* HET mice in which exon 21 of the *Shank3* gene was targeted (Kouser et al., 2013). We found that abundance of *Shank3* transcripts was also significantly increased in this line (Figure 1B). Meanwhile, RNA-sequencing analyses for the whole brain of KO mice in which exons 6–7 of* Shank2*, another member of the *Shank* gene family, were targeted (Chung et al., 2019), showed a decrease in total *Shank2* transcripts and normal *Shank3* transcripts (Figure 1B), thus suggesting that an increase in *Shank3* transcripts may be specific to *Shank3* mutant mice with partial deletions of *Shank3* exons.

To directly validate the changes in *Shank3* transcripts in detail, we performed qRT-PCR analyses on four different brain regions (hippocampus, cortex, striatum, and cerebellum) from adult (postnatal day 70–84) *Shank3B* HET and KO mice, and their WT littermates. We used nine primer sets targeting different exons along the *Shank3* gene (Figure 1C). Furthermore, we performed qRT-PCR experiments on the brain regions in adult *Shank3-*overexpressing TG mice and their WT littermates as a control (Han et al., 2013b; Lee et al., 2017b; Jin et al., 2018a,c). As expected, expression of exons 13–16 (i.e., the deleted exons) decreased by 50% and 100% in the four brain regions of *Shank3B* HET and KO mice, respectively (Figure 1C). Moreover, the C-terminal exons (exons 19–22) showed decreased expression in the cortex and cerebellum from *Shank3B* HET and KO mice when compared to WT mice. However, expression levels of the N-terminal exons (exons 1–12) were unexpectedly and significantly increased in the hippocampus, cortex, and striatum of *Shank3B* HET and KO mice (Figure 1C). These N-terminal exons were expressed at normal levels in the cerebellum of *Shank3B* HET and KO mice. In contrast, all the examined *Shank3* exons were expressed at higher levels in the four brain regions of *Shank3* TG mice compared to WT mice (Figure 1C). Increased expression of N-terminal *Shank3* exons was also observed in the cortex and striatum, but not in the cerebellum (with the exception of exons 11–12), of juvenile (postnatal day 21–28) *Shank3B* HET and KO mice (Figure 1D). Figure 1E summarizes the qRT-PCR analyses.

**Figure 2:**
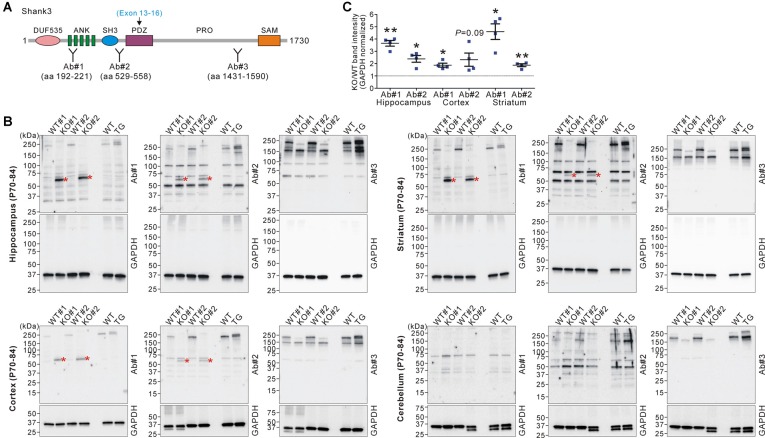
Western blot validation of expression of a small Shank3 isoform in the brain regions of *Shank3B* KO mice. **(A)** Targeted Shank3 regions of the antibodies (Ab#1~3) are indicated. Ab, antibody. Note that the deleted exons (13–16) in *Shank3B* mutant mice encode the PDZ domain of Shank3. **(B)** Western blot detection of Shank3 proteins by domain-specific Shank3 antibodies from whole lysates of the hippocampus, cortex, striatum, and cerebellum of adult WT, *Shank3B* KO, and *Shank3* TG mice. Note that a ~60 kDa band (asterisk) was detected in the hippocampus, cortex, and striatum of KO, but not WT and TG, mice by N-terminal antibodies #1 and #2. Also note that, in the cerebellum, there is no such KO-specific ~60 kDa band detected. **(C)** Quantification of fold-increases of the ~60 kDa band in the *Shank3B* KO brains compared to the WT brains (*n* = 4 animals per genotype). Data are presented as mean ± SEM. *P < 0.05; **P < 0.01 (unpaired two-tailed Student’s *t*-test).

Next, we investigated whether the increased *Shank3* transcripts in *Shank3B* KO mice were translated to produce Shank3 proteins. We performed western blot analyses on the brain lysates from WT, KO, and TG mice using three different domain-specific Shank3 antibodies (Lee et al., 2015; Figure 2A). Notably, antibodies against the N-terminal regions (Ab#1 and Ab#2), but not against the C-terminal region (Ab#3), of Shank3 detected a ~60 kDa protein band in the hippocampus, cortex, and striatum of *Shank3B* KO mice (Figures 2B,C). Importantly, the band was not detected in the WT and TG brains. The protein size approximately corresponded to the number of amino acids (~540 residues) encoded by exons 1–12 of the *Shank3* gene. These results suggest that the ~60 kDa Shank3 protein detected in the hippocampus, cortex, and striatum of *Shank3B* KO mice was likely translated from the increased *Shank3* exon 1–12 transcripts in the mice. Consistently with this interpretation, we did not detect the KO-specific ~60 kDa protein in the cerebellum (Figure 2B) where expression of the N-terminal *Shank3* exons was normal in *Shank3B* KO mice (Figure 1C). Nevertheless, our western blot results should be considered cautiously and require further validation with additional, if available, Shank3 domain-specific antibodies because there were multiple faint bands detected by the antibodies.

In this study, we observed an unexpected increase in *Shank3* transcripts in the brain regions in *Shank3* HET and KO mice having partial deletions of particular exons. The increase in *Shank3* transcripts was unlikely to be a non-specific outcome of chromosomal changes in the *Shank3* gene because it was observed in two different *Shank3* mutant mouse lines (i.e., *Shank3B* and *Shank3ΔC*) with different *Shank3* exonal deletions, and because it was not observed in the cerebellum of *Shank3B* mutant mice based on qRT-PCR. Moreover, the increase mainly occurred in the N-terminal but not C-terminal exons in *Shank3B* mutant mice, which suggests selective compensatory activation of upstream *Shank3* promoters in the process. Even so, it is not immediately clear how loss of synaptic Shank3 leads to the activation of *Shank3* promoters. One candidate player for this feedback mechanism is β-catenin, which, upon loss of synaptic Shank3, translocates from the synapse to the nucleus to induce histone deacetylase 2 (HDAC2)-dependent transcriptional changes (Qin et al., 2018). Whether β-catenin directly binds to the upstream *Shank3* promoters to induce their transcription remains to be validated.

Any functional effect of the increased *Shank3* transcripts in *Shank3* mutant mice also remains to be investigated. Our western blot analyses suggest that *Shank3B* KO mice produce a small Shank3 isoform, possibly having the N-terminal DUF535, ANK, and SH3 domains. This short isoform may function in a dominant-negative manner by sequestering some N-terminal Shank3 interactors, and thereby contributing to synaptic changes observed in the KO mice. Indeed, functional roles of the N-terminal part of Shank3 have been revealed by several studies (Hayashi et al., 2009; Cochoy et al., 2015; Lilja et al., 2017; Hassani Nia and Kreienkamp, 2018).

Regardless of the detailed underlying mechanisms and potential functional effects, our finding provides another layer of complexity with respect to regulating *Shank3* expression in the brain. We suggest that this may also contribute to phenotypic heterogeneity between *Shank3* mutant mouse lines with partial deletions of exons. For example, even with activation of the upstream *Shank3* promoters, no or minimal *Shank3* transcript increase may be observed in mutant mouse lines having deletions in the N-terminal *Shank3* exons (Bozdagi et al., 2010; Peca et al., 2011; Wang et al., 2011). Meanwhile, the increased *Shank3* transcripts in *Shank3ΔC* mice in which exon 21 of the *Shank3* gene was targeted (Kouser et al., 2013), may produce longer Shank3 protein isoforms than the ~60 kDa isoform detected in *Shank3B* KO mice. Comprehensive qRT-PCR validation of exon-specific *Shank3* transcripts and western blot analyses using domain-specific Shank3 antibodies in the brain regions of different *Shank3* mutant mouse lines are necessary to confirm this intriguing hypothesis.

## Data Availability Statement

The datasets generated for this study are available on request to the corresponding author.

## Ethics Statement

The animal study was reviewed and approved by Committee on Animal Research at the Korea University College of Medicine (KOREA-2016-0096).

## Author Contributions

CJ, HRK, HK, YZ, YL, YK and KH designed and performed the experiments. HK and KH analyzed and interpreted the data. KH wrote the article. All authors have read and approved the manuscript.

## Conflict of Interest

The authors declare that the research was conducted in the absence of any commercial or financial relationships that could be construed as a potential conflict of interest.
