# Supplementary material for: Unexpected Compensatory Increase in Shank3 Transcripts in Shank3 Knock-Out Mice Having Partial Deletions of Exons
Source: Front Mol Neurosci. 2019 Sep 19;12:228. doi: 10.3389/fnmol.2019.00228 (PMC6761322; doi:10.3389/fnmol.2019.00228)
Supplement: Supplementary file 1 [file Data_Sheet_1.PDF]

Supplementary material

Unexpected compensatory increase in *Shank3* transcripts in *Shank3* knock-out mice having partial deletions of exons

Chunmei Jin, Hyae Rim Kang, Hyojin Kang, Yinhua Zhang, Yeunkum Lee, Yoonhee Kim and Kihoon Han

Supplementary figure

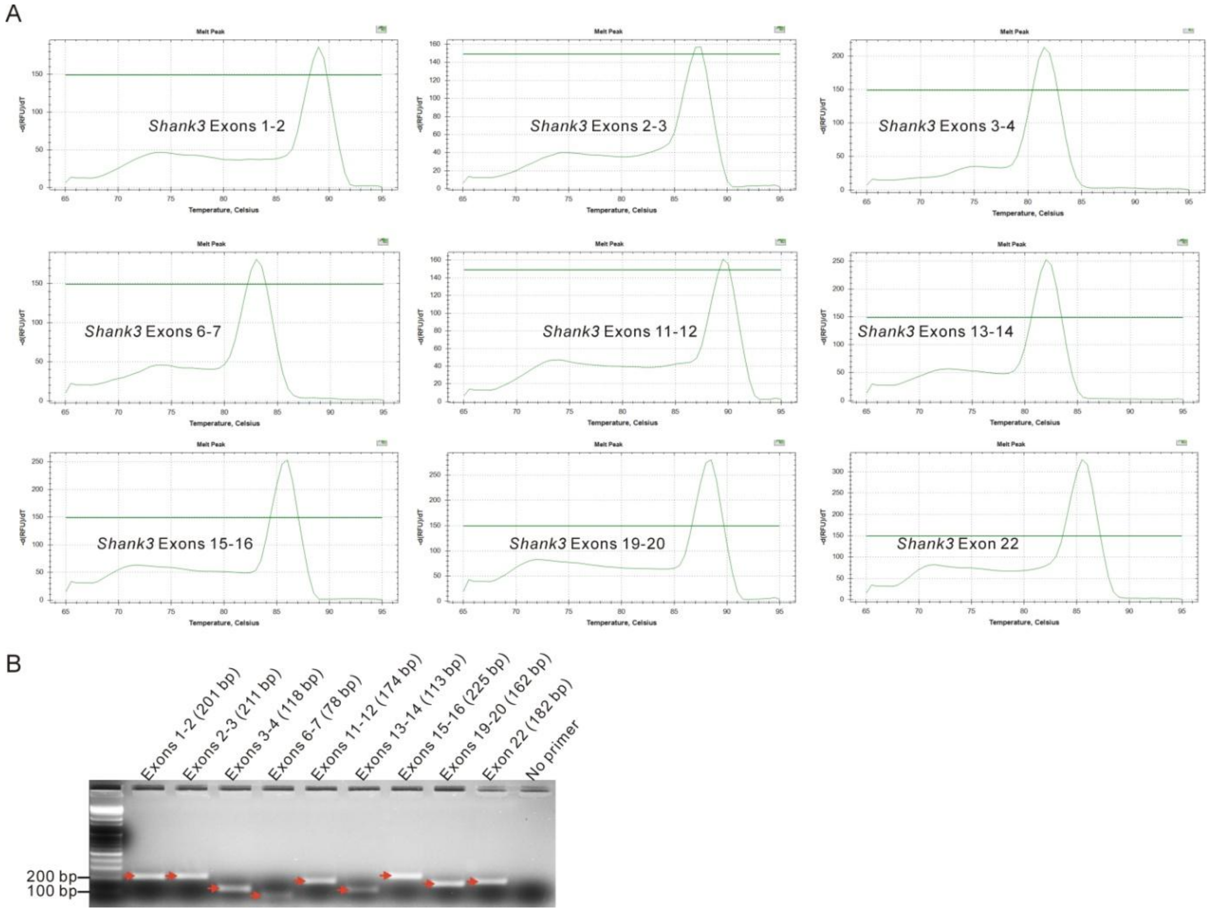

Figure S1. Confirmation of specificity of the *Shank3* primer sets. (A) Graphs showing the melting peaks of qRT-PCR reactions. Each primer set shows a sharp single peak. **(B)** Agarose gel image of the PCR products of qRT-PCR reactions. Each primer set produced a single band product (red arrow) with an expected size.
